# *Plasmodium falciparum pfhrp2* and *pfhrp3* Gene Deletions in Malaria-Hyperendemic Region, South Sudan

**DOI:** 10.3201/eid2901.220775

**Published:** 2023-01

**Authors:** Irene Molina-de la Fuente, María José Sagrado Benito, Laurence Flevaud, Janet Ousley, Harriet Akello Pasquale, Ahmed Julla, Abdirashid M. Abdi, Buai Tut Chol, Bakri Abubakr, Agustín Benito, Cristian Casademont, Carolina Nanclares, Pedro Berzosa

**Affiliations:** Institute of Health Carlos III, Madrid, Spain (I. Molina-de la Fuente);; Alcala University, Madrid (I.M. de la Fuente, A. Benito, P. Berzosa);; Médecins Sans Frontières, Barcelona, Spain (M.J.S. Benito, L. Flevaud, C. Casademont, C. Nanclares);; Médecins Sans Frontières, New York, New York, USA (J. Ousley);; National Malaria Control Program, Ministry of Health, Juba, South Sudan (H.A. Pasquale, A. Julla);; Médecins Sans Frontières, Juba (A.M. Abdi, B.T. Chol);; Médecins Sans Frontières, Nairobi, Kenya (B. Abubakr);; Centro de Investigación Biomedica en Red de Enfermedades Infecciosas, Madrid (A. Benito, P.J.B. Diaz)

**Keywords:** malaria, *Plasmodium falciparum*, histidine-rich protein, RDT, surveillance, parasites, diagnosis, molecular epidemiology, vector-borne infections, South Sudan

## Abstract

*Pfhrp2* and *pfhrp3* gene deletions threaten the use of *Plasmodium falciparum* malaria rapid diagnostic tests globally. In South Sudan, deletion frequencies were 15.6% for *pfhrp2*, 20.0% for *pfhrp3*, and 7.5% for double deletions. Deletions were approximately twice as prevalent in monoclonal infections than in polyclonal infections.

Histidine-rich protein 2 (HRP2) is the primary target of the *Plasmodium falciparum* rapid diagnostic tests (RDT) that are a cornerstone of malaria control efforts in the high-burden, low-resource contexts in which malaria mortality is most acute (*1*). Increasing prevalence of *P. falciparum* parasites that do not express HRP2 or its paralogue histidine-rich protein 3 (encoded by the *pfhrp2* and *pfhrp3* genes) are affecting the accuracy of the RDTs. Infections with *pfhrp2* deletions are missed by HPR2-based RDTs much of the time; infections with double deletions (missing both *pfhrp2* and *pfhrp3* genes) are invisible to RDTs and create false-negative results. Because these deletions represent an existential threat to recent gains made in malaria control, the World Health Organization (WHO) has emphasized the critical need for surveillance (*2*).

Malaria is a leading cause of illness and death in South Sudan (*3*), where insufficient malaria prevention activities and a lack of access to healthcare combine dangerously. Despite the geographic and strategic importance of South Sudan in East and Central Africa, the only evidence of *pfhrp2* and *pfhrp3* deletions from the country come from a single report confirming their presence in 3 travelers to Australia (*4*). Accurate estimates of deletions could help responders delineate factors associated with deletions, predict future RDT needs, and clarify dynamics of false negativity rates in South Sudan overall.

In 2019, in collaboration with the South Sudanese Ministry of Health, Médecins Sans Frontières began a seasonal malaria chemoprophylaxis campaign combined with an assessment of molecular markers of antimalaria drug resistance in Yambio County, a malaria-endemic region of Western Equatoria State. We describe the frequency of *pfhrp2* and *pfhrp3* and double deletions in this clinical cohort, as well as the association between deletions, demographic factors, and infection characteristics in South Sudan. This study was approved by the internal ethics review board at Médecins Sans Frontières and by the South Sudan Research Ethics Committee. All participants provided informed consent.

## The Study

We analyzed finger-prick blood samples collected in 9 villages in Yambio at the end of the malaria peak (January–February 2020) from persons >6 months of age with symptomatic malaria infection positively diagnosed by pan-pLDH–based RDT (CareStart Malaria PAN [pLDH] Ag RDT; Access Bio, https://accessbio.net). We performed malaria confirmation and speciation of 594 dried blood spot samples by multiplex PCR (*5*). Confirmed *P. falciparum* samples with high DNA quality (n = 518) underwent genotyping and molecular analysis for deletions in exon 2 of *pfhrp2* and *pfhrp3* (*5*) (Appendix). Demographic information was collected for all samples (Appendix). We defined multiplicity of infection (MOI) as the number of parasitic genotypes per infection and analyzed for a random subsample (n = 419) by amplifying *P. falciparum* merozoite surface protein 1 and 2 genes (*pfmsp1*, *pfmsp2*) (*6*). We defined monoclonal infection as the detection of a single PCR fragment for each locus and polyclonal infection as the detection of >1 PCR fragment for >1 locus.

**Figure 1:**
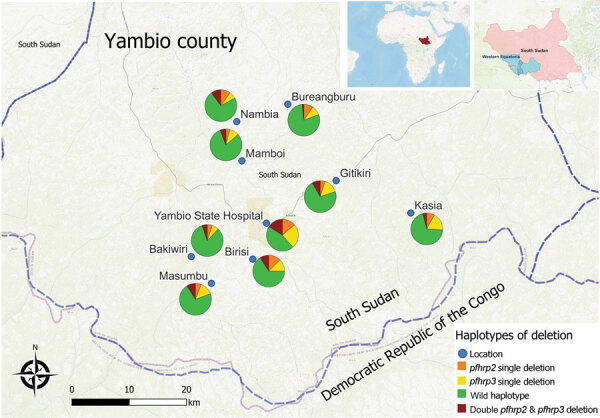
Frequencies of *Plasmodium falciparum* single and double *pfhrp2* and *pfhrp3* deletions in malaria-hyperendemic region, South Sudan. Color represents the type of deletion and proportion of each type of deletion and genotype. Open source QGIS software (https://www.qgis.org) was used to map sample collection locations. Inset map shows locations of the study area in South Sudan and of South Sudan in Africa.

Overall deletion frequency (including samples with both single and double deletions) among 518 genotyped PCR-positive samples was 15.6% for *pfhrp2* and 20.0% for *pfhrp3* (Table 1). Double deletions were found in 7.5% of isolates; patients at Yambio State Hospital had nearly twice the rate of double deletions (15.9%) as patients at all other sites (Figure 1). In 7/9 study sites, >10% of samples did not amplify *pfhrp2*; >5% of isolates were double-deleted in nearly half (4/10) of sites. *Pfhrp2* deletion rates in South Sudan were as high as or higher than the country’s immediate neighbors, where reported deletion rates from specific sites have varied from 26% in Ethiopia to 19% in Kenya, 6% in the Democratic Republic of the Congo, 3% in Uganda, and <1% in Sudan (*7*–*11*).

**Table 1:** Frequency of *pfhrp2 and pfhrp3* deletion by geographic origin of samples, South Sudan*

Location	No isolates included	Overall *pfhrp2* deletion			Overall *pfhrp3* deletion	
		No.	Frequency, % (95% CI)		No.	Frequency, % (95% CI)
All sites	518	81	15.64 (12.62–19.06)		104	20.08 (16.71–23.79)
Kasia	50	6	12.00 (4.53–24.31)		11	22.00 (11.53–35.96)
Yambio State Hospital	44	13	29.55 (16.76–45.20)		18	40.91 (26.34–56.75)
Birisi	56	12	21.43 (11.59–34.44)		12	21.43 (11.59–34.44)
Bureangburu	62	7	11.29 (4.66–21.88)		7	11.29 (4.66–21.88)
Bakiwiri	58	6	10.34 (3.89–21.17)		7	12.07 (4.99–23.30)
Gitikiri	60	8	13.33 (5.94–24.59)		14	23.33 (13.38–36.04)
Nambia	70	14	20.00 (11.39–31.27)		12	17.14 (9.18–28.03)
Mamboi	51	5	9.80 (3.26–21.41)		7	13.73 (5.70–26.25)
Masumbu	67	10	14.93 (7.40–25.74)		15	22.39 (13.11–34.22)
p value by χ^2^ test		0.108 (13.089)			0.014 (18.988)	

*Deletion frequency was calculated by dividing confirmed deletions by all confirmed *Plasmodium falciparum* samples included for analysis. *pfhrp2* and *pfhrp3* deletion frequency includes both single and double deletions. All analyses used a 95% CI and a p value of <0.05 for statistical significance.

Monoclonality was the only factor significantly associated with both *pfhrp2* and *pfhrp3* deletions and double deletions (Table 2). Even so, the frequency of deletion among polyclonal infections was higher than expected. Severe malaria cases exhibited significantly more *pfhrp3* and double deletions than uncomplicated infections. Patients >14 years of age were more likely to harbor deletions than were patients <5 and 5–14 years of age, although the difference was significant only for *pfhrp3* (Table 2).

**Table 2:** Association of age, sex, MOI, severity of infection, and previous seasonal malaria chemoprophylaxis with *pfhrp2* and *pfhrp3* deletions in malaria-hyperendemic region, South Sudan*

Characteristic	Total no.	*pfhrp2* deletion			*pfhrp3* deletion			*pfhrp2/3* double deletion			Wild-type	
		No.	Frequency, % (95% CI)		No.	Frequency, % (95% CI)		No.	Frequency, % (95% CI)		No.	Frequency, % (95% CI)
Age, y												
<5	159	28	17.61 (12.03–24.44)		29	18.24 (12.49–24.98)		14	8.81 (4.87–14.25)		117	73.58 (66.02–80.25)
5–14	196	24	12.24 (8.00–17.67)		30	15.31 (10.57–21.12)		10	5.10 (2.47–9.18)		152	77.55 (71.06–83.19)
>14	163	30	18.40 (12.77–25.21)		45	27.61 (20.90–35.14)		15	9.20 (5.24–14.72)		103	63.19 (55.29–70.60)
p value (χ^2^)		0.238 (2.874)			0.012 (8.875)			0.261 (2.686)			0.009	
Sex												
F	271	44	16.24 (12.05–21.18)		60	22.14 (17.34–27.56)		24	8.86 (5.76–12.89)		191	70.48 (64.66–75.84)
M	247	37	14.98 (10.77–20.05)		44	17.81 (13.25–23.17)		15	6.07 (3.44–9.82)		181	73.79 (67.30–78.69)
p value (χ^2^)		0.694 (0.154)			0.219 (1.507)			0.231 (1.438)				0.542
MOI												
1: monoclonal	116	26	22.41 (15.19–31.09)		37	31.90 (23.55–41.19)		14	12.07 (6.76–19.42)		102	87.93 (80.58–93.24)
>2: Polyclonal	303	43	14.29 (10.54–18.76)		46	15.28 (11.41–19.85)		15	4.98 (2.82–8.09)		288	95.05 (91.97–97.20)
p value (χ^2^)		0.001 (9.881)			<0.001 (14.754)			0.010 (6.598)				0.019
Severity												
Uncomplicated	472	68	14.41 (11.36–17.90)		82	17.37 (14.06–21.10)		31	6.57 (4.51–9.19)		359	73.56 (71.95–79.84)
Complicated	30	8	26.67 (12.28–45.89)		14	46.67 (28.34–65.67)		5	16.67 (5.64–34.72)		13	43.33 (25.46–62.57)
p value (χ^2^)		0.087 (2.937)			<0.001 (14.031)			0.051 (3.819)				<0.001
Seasonal malaria chemoprophylaxis for children <5 y												
Yes	137	24	17.52 (11.56–24.94)		22	16.06 (10.35–23.24)		11	8.03 (4.08–13.91)		102	74.45 (66.30–81.52)
Not	22	3	13.64 (2.91–34.91)		7	31.82 (13.86–54.87)		3	13.63 (2.91–34.91)		15	68.18 (45.13–86.14)
p value (χ^2^)		0.652 (0.203)			0.075 (3.157)			0.742 (0.389)			0.720 (0.129)	

*Variables were considered categorical variables and the association between them and deletions were assessed using χ^2^ testing. Deletions for *pfhrp2* and *pfhrp3* include both single and double deletions. All analyses used a 95% CI and a p value of <0.05 for statistical significance. MOI, multiplicity of infection.

Unequal deletion distribution between proximate geographic zones has been previously reported (*9*,*10*), and variability in double deletion rates by study site in Yambio was notable, suggesting possible hot spots. However, the higher rates seen at Yambio State Hospital could also be explained by its significantly higher proportion of monoclonal infections (Appendix). The small sample sizes at individual sites leave conclusions about hot spots unsettled, but they underscore the importance of realistic malaria control strategies targeted to the local molecular marker landscape.

**Figure 2:**
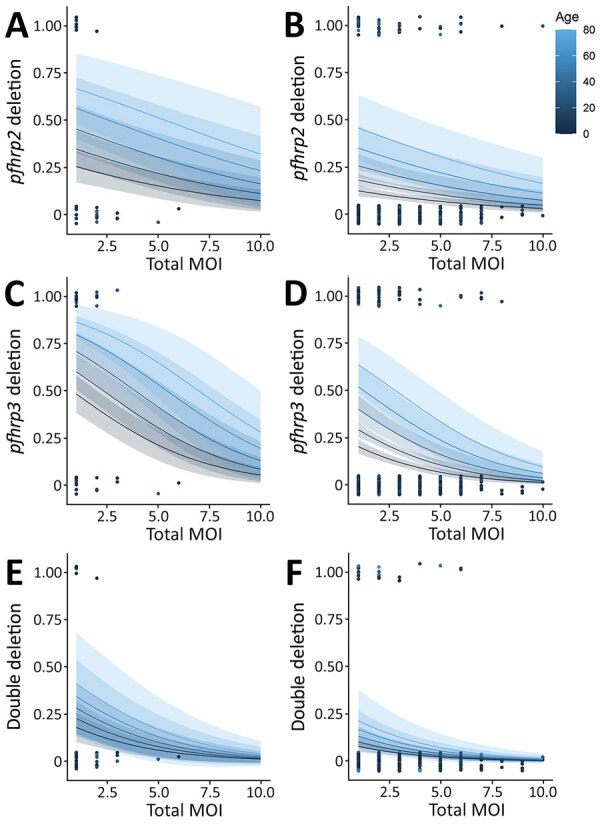
Multivariable regression model of *Plasmodium falciparum* *pfhrp2* deletions (A), *pfhrp3* deletions (B), and double deletions (C) in malaria-hyperendemic region, South Sudan. Panels A, C, and E indicate uncomplicated malaria; panels B, D, and F, severe malaria. The models combined 2 continuous variables: age of the patient, represented with different colors, and total MOI, represented in x-axis, with the binary response variable (presence of deletion). Probability of deletion (y-axis) was considered a binary outcome variable. The quality of the model was evaluated by the likelihood ratio method. The model was significant (p value<0.01) for *pfhrp2* and *pfhrp3* deletion and *pfhrp2* and *pfhrp3* double deletion. Each dot represents 1 sample. MOI, multiplicity of infection.

We used a statistically significant multivariable logistic regression model for total MOI, infection severity, and patient age factors for *pfhrp2*, *pfhrp3* and double deletions (Figure 2). Age was the only significant predictor of *pfhrp2* deletions (after adjusting for MOI and severity of infection), whereas age, MOI, and clinical severity were all predictors for *pfhrp3* deletions. Only MOI was a significant predictor of double deletions (Appendix).

Most research surrounding the interaction between MOI and *pfhrp2* and *pfhrp3* deletions concludes that polyclonal infections mask deletions and lead to underestimates in deletion prevalence (*12*). Our results support this conclusion, finding lower MOI in Yambio associated with most deletions. In high-transmission settings, younger persons tend to have higher MOIs (*13*). We also found lower MOI and older patient age associated with deletions, contrasting with studies that have linked deletions to lower age but failed to consider MOI as a confounder (*9*).

In addition, the fitness-cost of deletions (the effects on the parasite after losing 1 of its proteins) could be another way that age, disease severity, and deletion risk interact, because milder disease has previously been associated with *pfhrp2* deletion (*14*) and persons acquire immunity against *P. falciparum* as they age (*13*). In this area, our cohort breaks with consensus, finding deletions more commonly in complicated malaria patients. We believe this difference might reflect the difficulties of diagnosing febrile disease in South Sudan, where the signs of severe malaria might be caused by other undetected infections.

Sample collection in this study occurred at the end of the high-intensity malaria transmission season, when potentially high parasitic diversity but low prevalence could favor spread of gene-deleted organisms, making deletions easier to detect (*14*). When interpreting *pfhrp2* deletion surveillance, the transmission period should be considered (*9*,*15*).

This study was limited because it was a secondary analysis of a study of molecular markers of antimalarial drug resistance and did not follow WHO protocols for *pfhrp2* and *pfhrp3* deletion surveillance. Consequently, the precise prevalence of *pfhrp2* and *pfhrp3* gene deletions causing false-negative results on RDTs in South Sudan was not generated to assess whether it is within the 5% threshold established by WHO (*2*). We also exclusively used pan *p*-LDH RDT–positive samples, preventing us from evaluating the effects of deletions on malaria diagnoses.

## Conclusions

Characterizing *pfhrp2* and *pfhrp3* deletions is critical to designing effective public health strategies for malaria control. This study describes these deletions in a clinical cohort in a country with little previous endemic evidence of *pfhrp2* and *pfhrp3* deletion. Monoclonal infections were a principal predictor of deletions. We identified high levels of single and double deletions of *pfhrp2* and *pfhrp3*, which if more widely present in this or other regions of South Sudan, could s eriously jeopardize HRP2*-*based RDT effectiveness moving forward. Future studies should be designed according to WHO protocol to produce precise estimates to measure the risk those deletions pose in South Sudan. Local variation in prevalence suggests the potential for deletion hotspots within the country and should be considered when designing malaria control strategies. 

Appendix Additional information about *Plasmodium Falciparum pfhrp2* and *pfhrp3* gene deletions in malaria-hyperendemic region, South Sudan
