# Supplementary material for: Plasmodium falciparum pfhrp2 and pfhrp3 Gene Deletions in Malaria-Hyperendemic Region, South Sudan
Source: Emerg Infect Dis. 2023 Jan;29(1):154–9. doi: 10.3201/eid2901.220775 (PMC9796199; doi:10.3201/eid2901.220775)
Supplement: Appendix — Additional information about Plasmodium Falciparum pfhrp2 and pfhrp3 gene deletions in malaria-hyperendemic region, South Sudan [file 22-0775-Techapp-s1.pdf]

Article DOI: https://doi.org/10.3201/eid2901.220775

Plasmodium falciparum pfhrp2 and *pfhrp3* Gene Deletions in Malaria-Hyperendemic Region, South Sudan

Appendix

Appendix Table 1. Haplotypes of *pfhrp2 and pfhrp3* deletion by geographic origin of the samples*

Location	Total samples	Isolates Included, N	Single - *pfhrp2* deletion		Single – *pfhrp3* deletion		*pfhrp2* and *pfhrp3* double deletion		Wild-type parasites	
			n	F (CI 95%)	n	F (CI 95%)	n	F (CI 95%)	n	F (CI 95%)
All sites	594	518	42	8.11% (5.91 – 10.80)	65	12.54% (9.82 – 15.71)	39	7.53 (5.41 – 10.15)	372	71.81 (67.73 – 75.65)
Kasia	60	50	4	8.00% (2.22 – 19.23)	9	18.00% (8.58 - 31.44)	2	4.00 (0.49 - 13.71)	35	70.00 (55.39 – 82.14)
Yambio State Hospital	49	44	6	13.64% (5.17 – 27.35)	11	25.00% (13.19 – 40.34)	7	15.91 (6.64 – 30.07)	20	45.45 (30.39 – 61.15)
Birisi	62	56	7	12.50% (5.18 – 24.07)	7	12.50% (5.18 – 24.07)	5	8.93 (2.96 – 19.62)	37	66.07 (52.19 – 78.19)
Bureangburu	68	62	6	9.68% (3.63 - 19.88)	6	9.68% (3.63 - 19.88)	1	1.61 (0.04 - 8.66)	49	79.03 (66.82 – 88.34)
Bakiwiri	63	58	3	5.17% (1.08 – 14.38)	4	6.70% (1.91 – 16.73)	3	5.17 (1.08 – 14.38)	48	82.76 (70.57 – 91.41)
Gitikiri	66	60	3	5.00% (1.04 – 13.92)	9	15.00% (7.10 – 26.57)	5	8.33 (2.76 – 18.38)	43	71.67 (58.56 – 82.55)
Nambia	79	70	7	10.00% (4.12 – 19.52)	5	7.14% (2.36 – 5.89)	7	10.00 (4.14 - 19.52)	51	72.86 (60.90 – 82.80)
Mamboi	57	51	2	3.92% (0.48 - 13.49)	5	9.80% (3.26 - 21.41)	3	5.88 (1.23 – 16.24)	41	80.39 (66.88 – 90.18)
Masumbu	90	67	4	5.97% (1.65 - 14.59)	9	13.43% (6.33 - 23.97)	6	8.96 (3.36 – 18.48)	48	71.64 (59.31 – 81.99)
p-value (χ^2)				0.55 (6.856)		0.137 (12.315)		0.256 (10.134)		0.006 (21.3)

*Deletion frequency was calculated by dividing confirmed deletions of each haplotype by all confirmed *P. falciparum* samples included for analysis. All analyses used a 95% confidence level and a p-value of ≤0.05 for statistical significance.

Appendix Table 2. Data about the samples included in *pfhrp2* and *pfhrp3* genotyping and in the subsample for *pfmsp1* and *pfmsp2* genotyping.

Characteristic	All samples	Samples included for *pfmsp1* and *pfmsp2* genotyping
Age group		
<5 y	159	110
5–14	196	144
>14	163	124
Sex		
F	271	201
M	247	177
pan–LDH diagnosis		
Negative	14	7
Positive	504	371
Malaria type		
Severe	30	29
Uncomplicated	472	349

Appendix Table 3. Association between *pfhrp2 and pfhrp3* deletion and population, parasite and infection factors

	Pfhrp2 deletion, n = 58		*Pfhrp3* deletion, n = 83		*Pfhrp2/3* deletion, n = 29	
Factor	OR (95% CI)	p value	OR (95% CI)	p value	OR (95% CI)	p value
Age	1.02 (1.00 - 1.04)	0.030	1.02 (1.01 – 1.04)	0.012	1.01 (0.98 – 1.04)	0.317
MOI	0.85 (0.71 – 1.00)	0.071	0.73 (0.61 – 0.87)	<0.001	0.73 (0.54 – 0.95)	0.034
Severity	2.37 (0.92 – 5.63)	0.058	3.64 (1.62 – 8.17)	0.002	2.54 (0.78 – 7.03)	0.091
Model	0.009		0.001		0.014	
Hosmer-Lemeshow Goodness of Fit *p value*	0.410		0.383		0.328	

*MOI was calculated first for each gene (*pfmsp1* and *pfmsp2*) as the total number of alleles found in any of the locus of each gene (K1, RO33, and MAD20 for *pfmsp1* and 3D7 and FC27 for *pfmsp2)*, then the MOI total was reported as the maximum MOI value from both *pfmsp1* and *pfmsp2*. The model was built with a sample size of 419 samples. Each model was also tested for accuracy using Hosmer-Lemeshow Goodness of Fit, the interpretation of this estimate stablished that if there are not significant difference between the estimated and the observed data, then the model fits well. MOI, multiplicity of infection; OR, odds ratio.

Appendix Table 4. Multiplicity of infection by location and group of age

Characteristic	N samples	Monoclonal infections (%)	Polyclonal infections (%)	MOI range	Mean MOI
Overall		27.82	72.18	1 - 10	1.93
Location					
Bakiwiri	51	31.37	68.63	1 - 9	1.95
Birisi	54	22.22	77.77	1 - 10	2.01
Gitikiri	58	22.42	77.59	1 - 6	2.01
Kasia	46	26.09	73.91	1 – 9	1.91
Masumbu	51	27.45	72.55	1 – 8	1.96
Mamboi	49	24.49	75.51	1 – 10	2.07
Nambia	70	20.00	80.00	1 - 7	2.14
Yambio State Hospital	40	57.50	42.50	1 - 5	1.42
p value		0.002			0.161
Age group, y					
<5	127	25.98	74.02	1 – 9	1.91
5–14	164	26.22	73.78	1 - 10	2.30
>14	128	31.35	68.75	1 - 10	1.81
p value		0.557			0.022

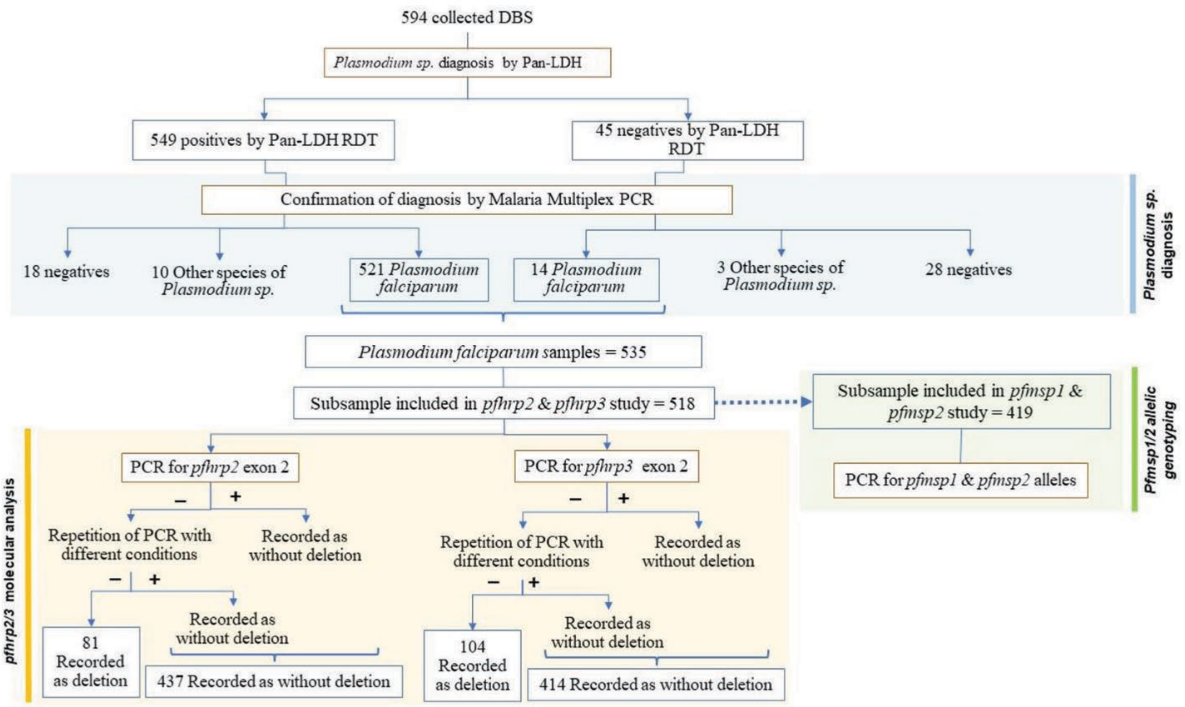

Appendix Figure. Methodological flow scheme. Pf-LDH – RDT diagnosis was confirmed by Nested Multiplex PCR, distinguishing *P. falciparum*, *P.vivax*, *P. ovale* and *P. malariae*. All the P. falciparum samples were amplified for *pfdhps, pfdhfr pfmdr1, pfcrt* and quantification by 18S, if they present any difficulty for amplification or a really low parasitemia they were excluded for deletion analysis. Finally, 17 samples were excluded and 518 included for deletion analysis. On the included *P. falciparum* samples, four independent PCRs were run to detect deletions in exon 1–2 and exon 2 of *pfhrp2* and *pfhrp3*. The deletion of any exon was confirmed with the absence of amplification after three PCR repetitions. Then a random subsample of 433 were included for allelic diversity analysis using *pfmsp1* and *pfmsp2* PCRs.
